# Efficacy of Zidovudine-Amikacin Combination Therapy *In Vitro* and in a Rat Tissue Cage Infection Model against Amikacin-Resistant, Multidrug-Resistant *Enterobacteriales*

**DOI:** 10.1128/spectrum.04843-22

**Published:** 2023-03-22

**Authors:** Wei-Tao Gong, Xiao-Jie Zhao, Gao-Ming Wang, Xiao-Lin Ma, Jian-An Huang

**Affiliations:** a Department of Pulmonary and Critical Care Medicine, The First Affiliated Hospital of Soochow University, Suzhou, Jiangsu, China; b Department of Pulmonary and Critical Care Medicine, Xuzhou Central Hospital, Xuzhou Clinical School of Xuzhou Medical University, Xuzhou, Jiangsu, China; c Department of Laboratory Medicine, The Affiliated Hospital of Xuzhou Medical University, Xuzhou, Jiangsu, China; d Department of Thoracic Surgery, Xuzhou Central Hospital, Xuzhou Clinical School of Xuzhou Medical University, Xuzhou, Jiangsu, China; e Department of Neurology, Xuzhou Central Hospital, Xuzhou Clinical School of Xuzhou Medical University, Xuzhou, Jiangsu, China; Huashan Hospital of Fudan University

**Keywords:** zidovudine, amikacin, combination, *Enterobacteriales*

## Abstract

Multidrug-resistant (MDR) *Enterobacteriales* infections have become an urgent global threat to public health. The aim of this study was to evaluate the efficacy of zidovudine-amikacin combination therapy *in vitro* and *in vivo*. Molecular characteristics and antibiotic resistance profiles of 53 amikacin-resistant MDR, extensively drug-resistant (XDR), or pan-drug-resistant (PDR) clinical isolates were examined via PCR and susceptibility testing. Checkerboard assays were performed for these 53 isolates to assess *in vitro* synergistic effects of the zidovudine-amikacin combination, and static time-kill experiments were performed for four XDR or PDR *Enterobacteriales* isolates. A Galleria mellonella model and a rat tissue cage infection model were established to assess *in vivo* synergistic effects. The *aac(6′)-Ib* gene was detected in 25 (47.2%) isolates, followed by *armA* in 5 (9.4%) isolates, *rmtB* in 27 (50.9%) isolates, and *rmtC* in 3 (5.8%) isolates. Checkerboard assays showed the synergy of this combination against 38 (71.7%) isolates. The time-kill assays further confirmed that zidovudine strongly synergized with amikacin against four XDR or PDR *Enterobacteriales* isolates. The Galleria mellonella model study showed that the survival benefit of zidovudine-amikacin combination therapy was significantly better than that of monotherapy for those four *Enterobacteriales* isolates. Furthermore, the rat tissue cage infection model study showed that zidovudine-amikacin combination therapy displayed more potent bactericidal activity than monotherapy after 3 and 7 days of treatment for the above four isolates. Our data support the idea that the zidovudine-amikacin combination could be a plausible alternative therapy against infections with amikacin-resistant MDR *Enterobacteriales*, especially with XDR and PDR *Enterobacteriales*.

**IMPORTANCE** Our study revealed for the first time that the zidovudine-amikacin combination shows a significant bactericidal effect against amikacin-resistant MDR, XDR, and PDR *Enterobacteriales*. Second, using *in vitro* and *in vivo* approaches, our study showed that zidovudine strongly synergized with amikacin against amikacin-resistant MDR *Enterobacteriales* isolates. Most importantly, with regard to survival benefit, pharmacokinetics, and bactericidal effects, our *in vivo* experiment demonstrated the effectiveness of zidovudine-amikacin.

## INTRODUCTION

*Enterobacteriales*, especially Klebsiella pneumoniae (K. pneumoniae) and Escherichia coli (E. coli), are a major cause of both community- and hospital-acquired infections worldwide (1). Over the last decade, the increasing prevalence of multidrug-resistant (MDR) and extensively drug-resistant (XDR) *Enterobacteriales*, especially carbapenem-resistant *Enterobacteriales*, such as New Delhi metallo-beta-lactamase-1 (NDM-1) carriers (2), has become an urgent global threat (3). Polymyxins are used as the last-resort treatment option to combat MDR and XDR *Enterobacteriales* infections (4). Unfortunately, rapid emergence of resistance to polymyxins was recently shown (5), and it is difficult to develop new effective antibiotics within a short time to combat bacterial antibiotic resistance (6). In such a difficult situation, the most promising therapeutic strategy is to reuse the existing antibiotics and restore the existing drugs potencies (7). Meanwhile, new or alternative combination regimens based on their *in vitro* synergistic activities are needed to overcome this problem (8).

Zidovudine (ZDV) is a thymidine analogue that is originally used to prevent and treat human immunodeficiency virus (HIV) infection (9). In addition, zidovudine can inhibit bacterial DNA replication by incorporation into the bacterial genome and subsequent DNA chain termination (10). Zidovudine has bactericidal activity against Gram-negative bacteria, including E. coli and K. pneumoniae (10, 11), and it has been shown to be efficacious in a rat model of systemic E. coli infection (12). Additionally, several *in vitro* and *in vivo* studies have shown synergistic antimicrobial activity of zidovudine in combination with several antibiotics, such as colistin, tigecycline, fosfomycin, carbapenems, and trimethoprim, against MDR *Enterobacteriales* (13–18). However, synergy between aminoglycosides and zidovudine has not yet been sufficiently investigated, especially in the setting of an *in vivo* amikacin (AMK)-resistant *Enterobacteriales* study.

Therefore, the aim of this study was to evaluate the efficacy of zidovudine-amikacin combination therapy against amikacin-resistant MDR *Enterobacteriales in vitro* and to assess the relationship between drug resistance genes and *in vitro* efficacy. Furthermore, we built a Galleria mellonella model and a rat tissue cage infection model both to simulate the immune system and *in vivo* pharmacokinetics (PK) and to validate the *in vitro* results.

## RESULTS

### *In vitro* susceptibility of zidovudine and amikacin against 53 *Enterobacteriales* strains.

The MICs of zidovudine ranged from 0.25 mg/L to more than 64 mg/L for all of the tested strains, with the MIC_50_ being 8 mg/L and MIC_90_ being 32 mg/L, which is consistent with the values reported for K. pneumoniae and E. coli by other studies (13, 16). Resistance to amikacin (≥128 mg/L) was found in all of the tested strains by the broth microdilution and agar dilution methods. There were no significant differences in the MICs obtained using the two methods.

### Resistance genes.

Amikacin resistance genes were screened for in all of the isolates via PCR (see Table S1 and Fig. S1 in the supplemental material). The *aac(6′)-Ib* gene was detected in 25 (47.2%) isolates, followed by *armA* in 5 (9.4%) samples. The *rmtB* gene was found in 27 (50.9%) isolates, including seven K. pneumoniae and 20 E. coli strains, whereas *rmtC* was detected in only three K. pneumoniae strains. All of the *Enterobacteriales* strains were negative for the *rmtA*, *rmtD*, *npmA*, and *aac(6′)-II* genes. Seven (13.2%) *Enterobacteriales* isolates did not possess these eight resistance genes. The *aac(6′)-Ib*, *armA*, *rmtB*, and *rmtC* amplicons were genetically sequenced and showed full agreement with the above-mentioned genes. The *aac(6′)-Ib* gene was found in association with either *armA*, *rmtB*, or *rmtC* in 13 *Enterobacteriales* strains.

### Checkerboard assay.

**FIG 1:**
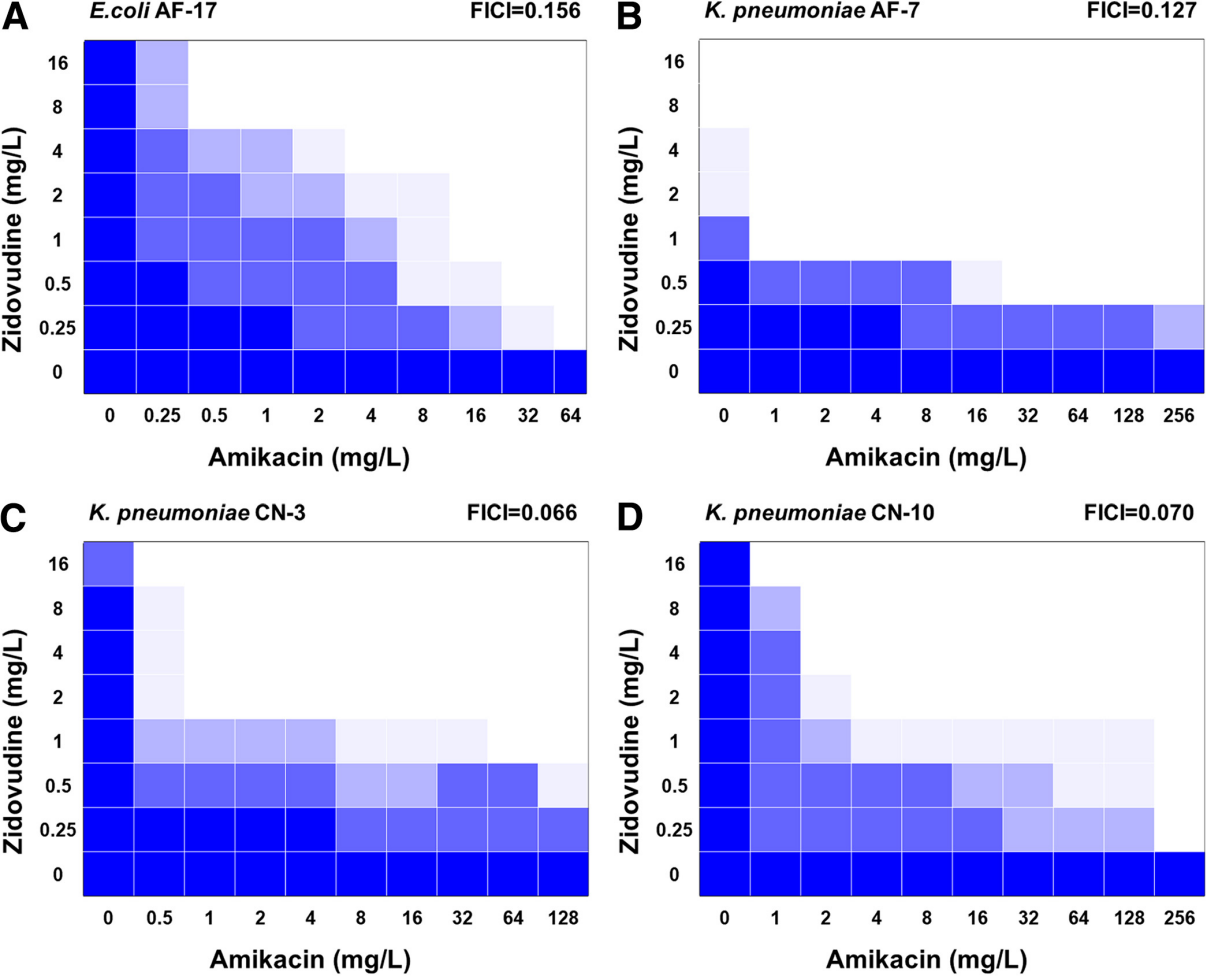
Synergistic activity between zidovudine and amikacin against E. coli AF-17, K. pneumoniae AF-7, K. pneumoniae CN-3, and K. pneumoniae CN-10. Mean OD_600_ values were recorded after 18 h of incubation. The dark blue regions represent higher bacterial density.

As shown in Table 1, the zidovudine-amikacin combination demonstrated synergy (fractional inhibitory concentration index [FICI] ≤ 0.5) against 38 (71.7%) of the isolates. No antagonism was observed for any of the isolates. For E. coli AF-17, K. pneumoniae AF-7, K. pneumoniae CN-3, and K. pneumoniae CN-10, the FICIs were 0.156, 0.127, 0.066, and 0.070, respectively (Fig. 1). In 40 (75.5%) of the isolates, amikacin restored the susceptibility of *Enterobacteriales* in combination with 2 mg/L zidovudine.

**TABLE 1:** Activities of zidovudine-amikacin

Strain group	Activity of the combination	FICI	No. (%) of strains
K. pneumoniae	Synergy	≤0.5	17 (77.27)
	No interaction	0.56–1	5 (22.73)
	Antagonism	>4	0

E. coli	Synergy	≤0.5	21 (67.74)
	No interaction	0.56–1	10 (32.26)
	Antagonism	>4	0

### Time-kill assay.

**FIG 2:**
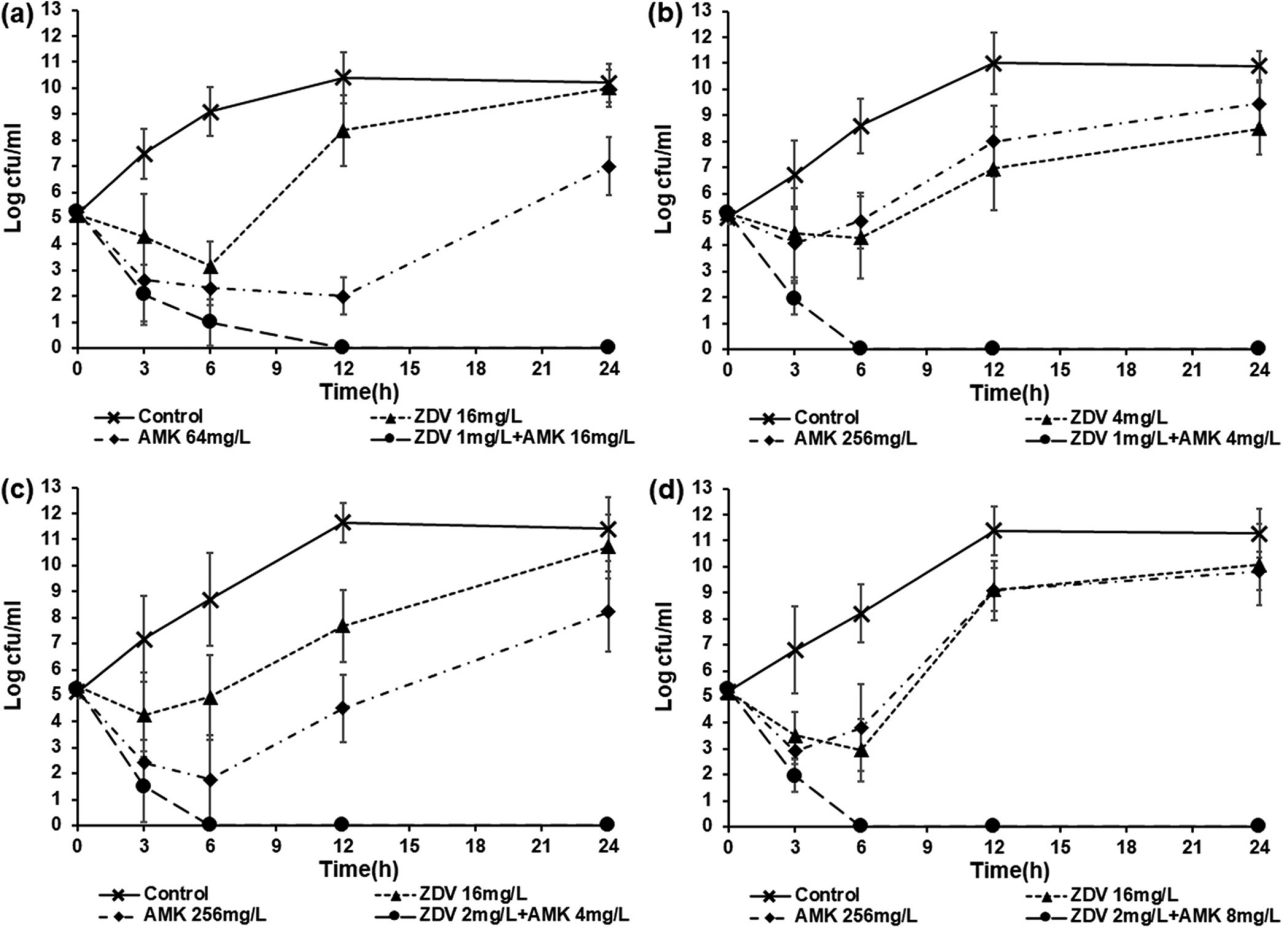
Time-kill curves showing the efficacy of zidovudine in combination with amikacin against E. coli AF-17 (a), K. pneumoniae AF-7 (b), K. pneumoniae CN-3 (c), and K. pneumoniae CN-10 (d).

The efficacy of the zidovudine-amikacin combination was also assessed via time-kill assay against E. coli AF-17, K. pneumoniae AF-7, K. pneumoniae CN-3, and K. pneumoniae CN-10 (Fig. 2). Both 1/2× MIC zidovudine monotherapy and 1/2× MIC amikacin monotherapy resulted in an initial decrease in bacterial counts, but regrowth occurred between 6 h and 24 h for all four isolates. The zidovudine-amikacin combination resulted in rapid bactericidal activity, and no bacterial regrowth (below the limit of detection of <10 CFU/mL) was observed within 24 h for all four isolates.

### Galleria mellonella infection model.

**FIG 3:**
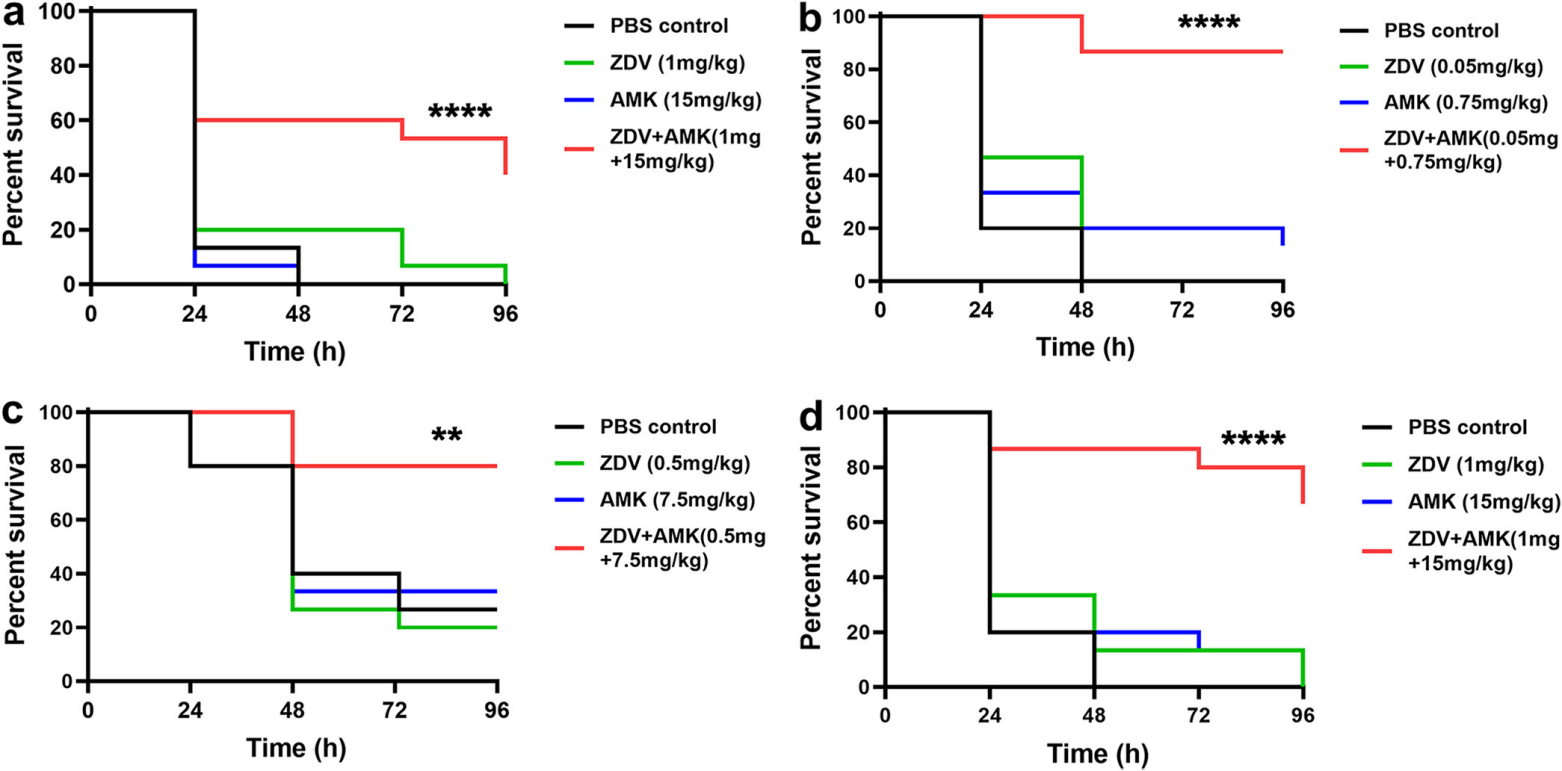
Survival curves showing the synergy between zidovudine and amikacin in G. mellonella larvae infected with E. coli AF-17 (a), K. pneumoniae AF-7 (b), K. pneumoniae CN-3 (c), and K. pneumoniae CN-10 (d). **, *P* < 0.01; ****, *P* < 0.0001.

To assess the therapeutic potential of zidovudine-amikacin *in vivo*, Galleria mellonella larva infection models infected by one E. coli strain (E. coli AF-17) and three K. pneumoniae strains (K. pneumoniae AF-7, K. pneumoniae CN-3, and K. pneumoniae CN-10) were constructed and employed (Fig. 3). In direct contrast to either of the two drugs alone, zidovudine-amikacin combination therapy showed a significant survival benefit for larvae infected with any of the strains (*P* < 0.01). For each of the four strains, no significant difference in larval survival was observed with either monotherapy group compared with control group (*P* > 0.12). In larvae infected with E. coli AF-17 (Fig. 3a), the combination therapy (1 mg/kg of body weight ZDV + 15 mg/kg AMK) ensured significantly greater survival than zidovudine or amikacin monotherapy (40% versus 0%, *P* = 0.002; 40% versus 0%, *P* < 0.001). In larvae infected with K. pneumoniae AF-7 (Fig. 3b), combination therapy at a low dose (0.05 mg/kg ZDV + 0.75 mg/kg AMK) also resulted in significantly greater survival than zidovudine or amikacin monotherapy (86.7% versus 0%, *P* < 0.001; 86.7% versus 13.3%, *P* < 0.001). In larvae infected with K. pneumoniae CN-3 (Fig. 3c), zidovudine and amikacin monotherapies resulted in 20% and 33.3% survival, respectively, whereas the combination therapy (0.5 mg/kg ZDV + 7.5 mg/kg AMK) ensured 75% survival (*P* < 0.001 and *P* = 0.008). The survival of larvae infected with K. pneumoniae CN-10 was also greater in those treated with the combination therapy than in those treated with zidovudine or amikacin monotherapy (80% versus 20%, *P* < 0.001; 80% versus 33.3%, *P* = 0.008) (Fig. 3d).

### Nephrotoxicity study and PK of zidovudine and amikacin in a rat tissue cage infection model.

**FIG 4:**
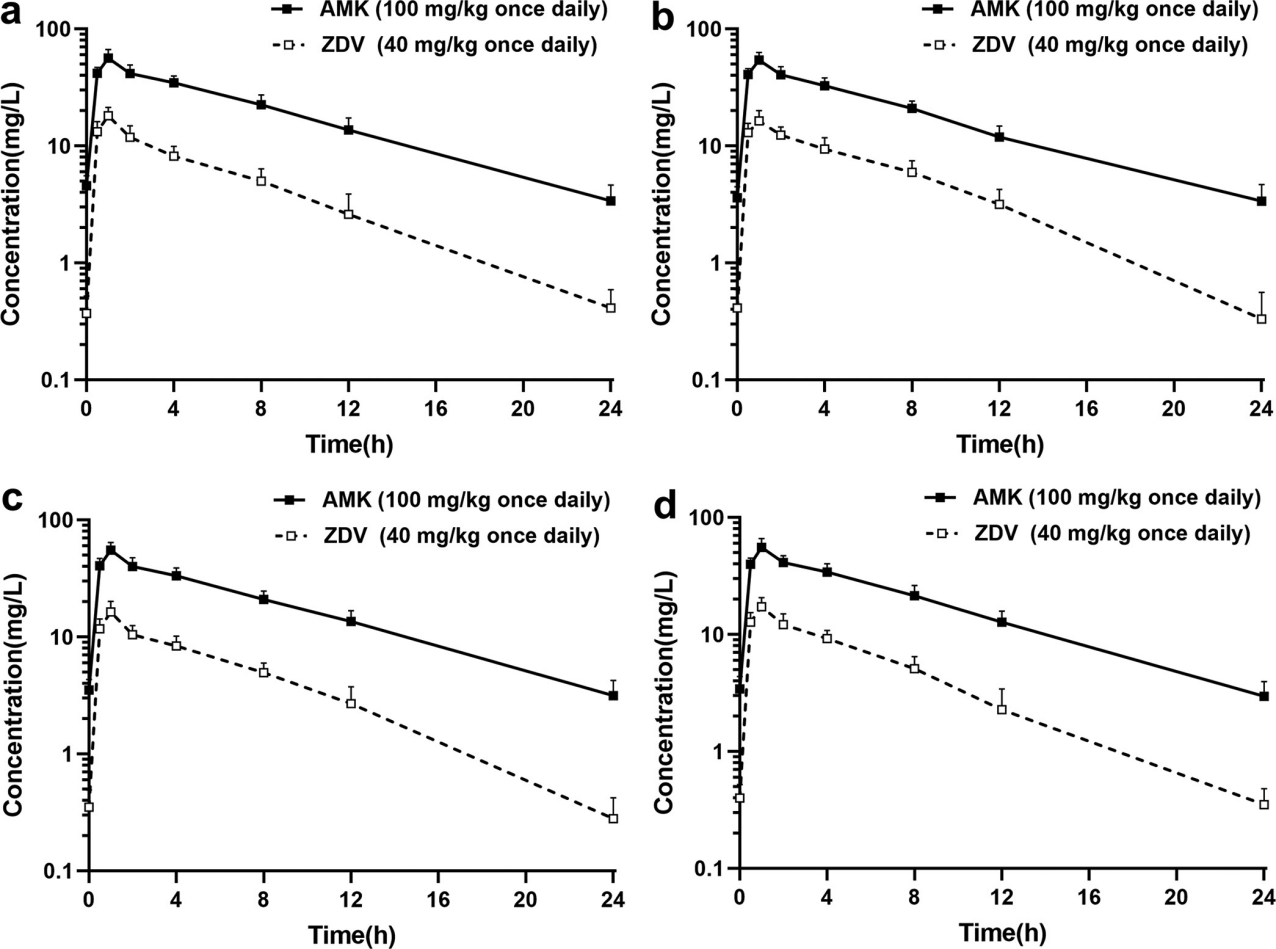
Concentration-time curves of amikacin (AMK) and zidovudine (ZDV) in the tissue cage fluid on day 7 of therapy for E. coli AF-17 (a), K. pneumoniae AF-7 (b), K. pneumoniae CN-3 (c), and K. pneumoniae CN-10 (d).

As shown in Fig. S2, no significant differences in reaching the nephrotoxicity endpoint were observed between the groups. The pharmacokinetic curves of zidovudine and amikacin in the rat tissue cage infection model after steady-state kinetics were obtained (Fig. 4). Interestingly, we found that zidovudine and amikacin in the tissue cages displayed comparable times to maximal drug concentration (*T*_max_) and similar concentration-time curves. The average peak and trough levels of zidovudine in the tissue cage fluid for the animals treated with 40 mg/kg once daily were 16.98 ± 3.23 and 0.34 ± 0.16 mg/L, respectively, and the average area under the concentration-time curve from 0 to 24 h (AUC_0–24_) was 107.45 ± 6.24 mg·h/L. The mean peak and trough levels for amikacin 100 mg/kg once daily were 55.3 ± 8.59 and 3.2 ± 1.05 mg/L, respectively, and the mean AUC_0–24_ was 433.3 ± 12.47 mg·h/L.

### Treatment outcome in a rat tissue cage infection model.

**FIG 5:**
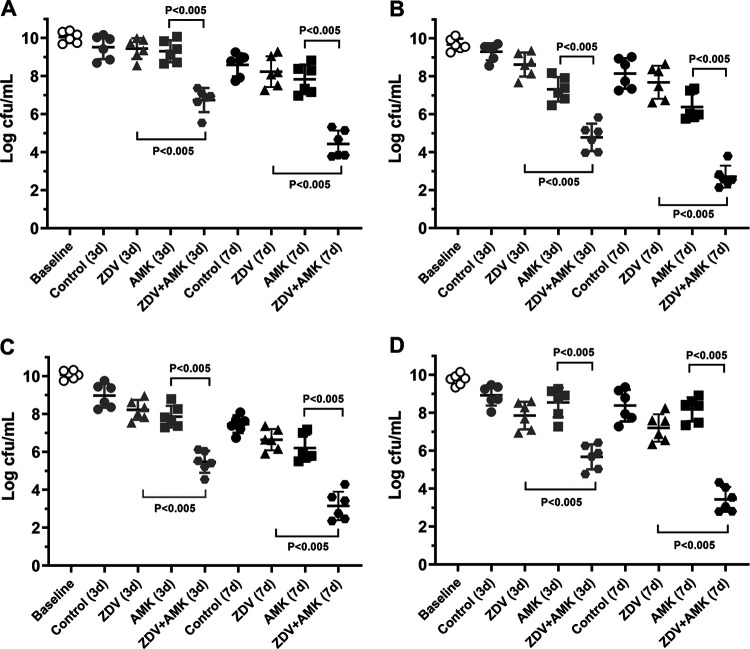
Bacterial burden of the tissue cage fluid for E. coli AF-17 (A), K. pneumoniae AF-7 (B), K. pneumoniae CN-3 (C), and K. pneumoniae CN-10 (D) after 3 days and 7 days of treatment.

The *in vivo* therapeutic potential of zidovudine-amikacin combination was studied using the rat tissue cage infection model. As shown in Fig. 5, for each isolate, no significant differences in bacterial counts were observed between the groups at the beginning of the treatment. The time-kill curves for the four strains (E. coli AF-17, K. pneumoniae AF-7, K. pneumoniae CN-3, and K. pneumoniae CN-10) displayed decreased bacterial numbers in both the treatment and control groups. The therapeutic potential of different therapies after 3 and 7 days of treatment was also analyzed. Compared with the control group, for all four strains, there was no >2-log reduction in CFU per milliliter with the monotherapies after 3 and 7 days of treatment. In contrast, the combination therapy displayed more potent bactericidal activity (>2 log CFU/mL) than monotherapies after 3 and 7 days of treatment for each of the four isolates (*P* < 0.005).

## DISCUSSION

In this study, we demonstrated that zidovudine synergized with amikacin against 38 (71.7%) MDR, XDR, or pan-drug-resistant (PDR) *Enterobacteriales* strains. The results of this study are in line with a previous study supporting the synergistic activity of zidovudine with gentamicin (19). Furthermore, we validated the synergy result using the Galleria mellonella model and the rat tissue cage infection model against four XDR or PDR *Enterobacteriales* strains.

Amikacin is effective against amikacin-susceptible MDR *Enterobacteriales* (20). However, amikacin resistance has been on the rise over the last decade worldwide (21). It is important to prolong the life of amikacin through effective combination therapy. In this study, we showed that in combination with zidovudine, the MIC of amikacin was significantly reduced and sensitivity to amikacin was restored. The efficacy of zidovudine-amikacin was confirmed with *in vitro* and *in vivo* time-kill assays, which dynamically monitored the bactericidal activities of the combination therapy over time. It was shown by the *in vitro* time-kill assays that amikacin was completely ineffective after 12 h of treatment but that the addition of zidovudine sustainably reduced bacterial counts, and no bacterial regrowth was observed. In the *in vivo* time-kill assays, no significant reduction was observed after 7 days of amikacin treatment compared with the control group, but zidovudine-amikacin combination therapy offered more potent bactericidal activity than monotherapies after 7 days of treatment for each of the four isolates. Collectively, the data suggest that further clinical trials of amikacin-zidovudine combination therapy may be used to develop this combination into an effective therapy against XDR and PDR *Enterobacteriales* strains.

Zidovudine has a safety profile with a recommended dose of 600 mg/daily over a patient’s lifetime due to its high selectivity over human DNA polymerase and short plasma half-life (22, 23). The most common toxic effects include myopathy, neutropenia, anemia, anorexia, hepatotoxicity, arthralgia, nausea, and vomiting (24). These adverse effects of zidovudine generally occur after long-term (>4-week) administration, but antibiotic treatment generally does not go beyond 14 days. The dosage of zidovudine could be increased to 2,400 mg/daily, since oral overdosage of 20 g does not cause bone marrow suppression or side effects in humans (25). In rats, the median lethal dose of zidovudine was found to be greater than 750 mg/kg intravenously (26). In this rat tissue cage infection model, the dosage was 40 mg/kg/day to simulate a 300-mg/day (6 mg/kg/day) dosage in humans via intravenous administration, and no potential additional side effects were observed in this study.

The pharmacokinetics of zidovudine has mainly been evaluated in HIV-infected individuals, following either oral or intravenous administration (22, 27). The peak plasma levels of zidovudine of 1 to 5 µg/mL are achieved after a single ingested dose of 200 to 300 mg zidovudine in HIV-infected individuals (28–30). Wattanagoon et al. reported that in healthy volunteers, the peak plasma concentration of zidovudine could achieve 4.8 µg/mL (17.98 µM) after oral administration of 300 mg (28). Wilde and Langtry reported that the plasma concentrations of zidovudine varied linearly depending on the dosage (31), and Antonello et al. inferred that the plasma concentrations of zidovudine could reach approximately 8 µg/mL after oral administration of 600 mg zidovudine (16). Mouse model systemic infection studies have shown that the plasma concentration of zidovudine was approximately 28.2 µg/mL after oral administration of zidovudine (50 mg/kg) (12). In this study, the concentration of zidovudine in the tissue cage fluids of the rats was 16.98 ± 3.23 µg/mL after intravenous administration of zidovudine (40 mg/kg once daily). The results of this study suggest that the therapeutically active levels of zidovudine in the tissue fluid can easily be exceeded after intravenous administration. In previous studies, zidovudine dosage regimens were designed for preventing and treating HIV infection, but there is no clear dosing regimen for bacterial infection. In this study, we chose the dosage based on the dosage for treating HIV infection and the administration method of combination therapy. This dosage regimen needs further validation in a clinical setting to enable translation of the dosage regimen to clinical benefits.

The mechanism of action of amikacin is to bind to the decoding aminoacyl-tRNA recognition site of the 16S rRNA that comprises the 30S ribosome subunit of bacteria and to subsequently inhibit protein synthesis (32). The modes of amikacin resistance in bacteria have been attributed to (i) enzymatic inactivation/modification of amikacin, (ii) modification or mutation of amikacin-binding site, (iii) decreased bacterial membrane permeability for amikacin, and (iv) augmented efflux of amikacin (33). Among them, the most prevalent mechanism of amikacin resistance in *Enterobacteriales* is inactivation of amikacin by aminoglycoside-modifying enzyme and 16S rRNA methylase (34). The main genes for resistance to amikacin include *armA*, *rmtA*, *rmtB*, *rmtC*, *rmtD*, *npmA*, *aac(6′)-Ib*, and *aac(6′)-II* (35). In this study, a total of 34 isolates positive for 16S rRNA methylase and 25 isolates positive for an aminoglycoside-modifying enzyme were identified. According to our results, there were no clear connections between these resistance genes and synergistic activity of zidovudine-amikacin combination against *Enterobacteriales*. It has been found that zidovudine is a nucleoside-reverse transcriptase inhibitor and that it substantially inhibits DNA synthesis of bacteria (36, 37). mRNA is generated from a strand of DNA and employed as a template for protein synthesis. Liu et al. concluded that zidovudine acts synergistically with tigecycline by inhibiting protein synthesis (37). The mechanism of the synergistic activity between zidovudine and amikacin is not clear and deserves further study.

This study had three limitations. First, the presence of foreign matter in the tissue cage infection model may have affected the bactericidal effect of the drugs. Second, the rat tissue cage infection model can simulate only local infection, not pneumonia and bloodstream infection. Last, since only amikacin-resistant isolates and only one fixed dosage of each drug were used in the rat tissue cage infection model, we were not able to fully evaluate the efficacy of the combination therapy against the susceptible isolates.

In conclusion, amikacin-zidovudine combination could be a plausible alternative therapy against infections with amikacin-resistant MDR strains of *Enterobacteriales*, especially against XDR and PDR *Enterobacteriales*. The optimal dosage and frequency and the mechanism of synergistic activity need further investigation to achieve a better clinical outcome.

## MATERIALS AND METHODS

### Bacterial strains.

A total of 53 clinical isolates of amikacin-resistant MDR *Enterobacteriales* (31 E. coli and 22 K. pneumoniae strains) were collected between 2019 and 2021 from two large general hospitals in China (Xuzhou Central Hospital, a 4,500-bed university hospital, and the Affiliated Hospital of Xuzhou Medical University, a 4,150-bed university hospital) (Table S1). Carbapenem resistance was defined as resistance to either meropenem or imipenem in line with the Clinical and Laboratory Standards Institute (CLSI) guidelines (38). Amikacin resistance was defined as a MIC of ≥64 mg/L in accordance with the CLSI guidelines (38). As shown in Table S1, all of these strains were tested for resistance to amoxicillin-clavulanate, piperacillin-tazobactam, ceftazidime, cefepime, aztreonam, imipenem, meropenem, amikacin, tobramycin, ciprofloxacin, and trimethoprim-sulfamethoxazole via antimicrobial susceptibility testing (Vitek 2 system; bioMérieux). In addition, 26 and 8 isolates of these *Enterobacteriales* were also resistant to tigecycline and colistin, respectively (Table S1).

### Antimicrobial susceptibility.

Susceptibility testing for amikacin was preliminarily performed in triplicate using the agar dilution method, as recommended by the CLSI guidelines (38), using a dose of 0.25 to 256 mg/L (Huarun Shuanghe Limin Pharmaceutical Co., Ltd., Jinan, China). A standard cation-adjusted Mueller-Hinton broth (CAMHB; Qingdao Hope Bio-Technology Co., Ltd., Qingdao, China) microdilution method was performed in accordance with the CLSI guidelines to confirm the MICs of amikacin and to evaluate the MICs of zidovudine (Shandong New Time Pharmaceutical Co., Ltd., Linyi, China).

### Genotypes.

Antibiotic resistance genes, including 16S rRNA methylase genes (*armA*, *rmtA*, *rmtB*, *rmtC*, *rmtD*, and *npmA*) and aminoglycoside-modifying enzyme genes [*aac(6′)-Ib* and *aac(6′)-II*] were screened for in all of the isolates via PCR as described previously (35, 39–41). The primers specific for these genes are listed in Table S2. PCR products were randomly selected, and the presence of the genes listed above was confirmed via sequencing.

### Checkerboard assay.

Combinations of amikacin and zidovudine were carried out in 96-well microtiter plates in triplicate on an initial inoculum of 5 × 10^5^ CFU/mL measured using the optical density at 600 nm (OD_600_). The maximum drug concentrations of amikacin and zidovudine tested were 256 mg/L and 64 mg/L, respectively. The synergistic effects were determined by calculating the FICI of the combination as follows (42): [(MIC of drug A tested in combination)/(MIC of drug A tested alone)] + [(MIC of drug B tested in combination)/(MIC of drug B tested alone)]. Synergy was defined as a FICI of ≤0.5, an absence of interaction was defined as a FICI of >0.5 and ≤4, and antagonism was defined as a FICI of >4 (42). E. coli ATCC 25922 was used as a quality control strain.

### Time-kill assay.

The efficacy of each drug and their combination against *Enterobacteriales* was assessed through a static time-kill experiment. We randomly selected four XDR or PDR *Enterobacteriales* isolates (E. coli AF-17, K. pneumoniae AF-7, K. pneumoniae CN-3, and K. pneumoniae CN-10) against which the zidovudine-amikacin combination showed synergy. For each isolate, a total of four dosage regimens—placebo control, 1/2× MIC zidovudine; 1/2× MIC amikacin, and zidovudine-amikacin—were tested at concentrations that were effective in the checkerboard assay. The experiment was carried out in 25 mL fresh CAMHB inoculated on an initial concentration of 5 × 10^5^ CFU/mL. Bacterial counts were evaluated by plating serial samples at 0, 3, 6, 12, and 24 h. Synergy was confirmed as a 2-log_10_ CFU/mL reduction at 24 h by the combination compared with the most active antibiotic alone (16, 43), in addition to a ≥2-log_10_ reduction compared with the 0-h count (44).

### Galleria mellonella infection model.

The toxicities of zidovudine, amikacin, and their combination and the optimum inoculum for larval killing were assessed as previously described (16, 45). After the verification of no detectable toxicity of the two drugs in G. mellonella larvae, the therapeutic potential of zidovudine-amikacin *in vivo* was experimentally assessed.

Galleria mellonella larvae (Huiyude Biotech Company, Tianjin, China) were injected with E. coli AF-17 (1.5 × 10^8^ CFU/larvae), K. pneumoniae AF-7 (1.5 × 10^8^ CFU/larvae), K. pneumoniae CN-3 (1.5 × 10^7^ CFU/larvae), or K. pneumoniae CN-10 (1.5 × 10^8^ CFU/larvae) via a 10-µL injection into the left proleg. The concentration of each strain was chosen based on the ability to produce staggered killing over 96 h. At 30 min after infection, G. mellonella larvae were randomly divided into four groups (*n* = 15 per group) and given a second injection with either sterile phosphate-buffered saline (PBS; control), zidovudine, amikacin, or the combination of amikacin and zidovudine in the right proleg. The larvae were incubated aerobically in petri dishes at 37°C. The survival rates of the larvae were recorded at 0, 24, 48, 72, and 96 h after inoculation. Larvae were recorded as dead if they did not respond to agitation or touch.

### Animals.

Pathogen-free, healthy male Sprague-Dawley rats weighing 250 ± 10 g were acquired from the Laboratory Animal Center of Xuzhou Medical University (Xuzhou, Jiangsu, China). This study was reviewed and approved by the Experimental Animal Welfare and Ethics Committee of the Xuzhou Medical University. All of the animal experiments were carried out in strict accordance with the Protocol for the Protection and Welfare of Animals.

### Rat tissue cage infection model and study groups.

Rat tissue cage infection models were built as previously described (46, 47). Rats were fully anesthetized via intraperitoneal injection of pentobarbital sodium (30 mg/kg), and a sterile, closed, polytetrafluoroethylene Teflon cylinder (4-mL volume) with regularly spaced 0.2-mm holes was implanted under the back skin. Penicillin (400,000 IU/kg of body weight) was intraperitoneally injected into the rats to prevent secondary infections for 3 days. Two weeks after implantation of the tissue cages, 100 µL of clear tissue cage fluid (TCF) was drawn by percutaneous aspiration to examine sterility. Subsequently, 200 µL of bacterial suspension (approximately 1.2 × 10^10^ CFU/mL) containing either E. coli AF-17, K. pneumoniae AF-7, K. pneumoniae CN-3, or K. pneumoniae CN-10 was injected into the tissue cages. After 2 days, 100 µL of TCF was withdrawn for quantitative bacterial load. A bacterial density of >1 × 10^9^ CFU/mL in TCF was defined as a successful rat tissue cage infection model.

The rats were randomly divided into four groups (*n* = 6), and received sterile saline, zidovudine, amikacin, or the combination of amikacin and zidovudine for 7 days via tail vein injection.

### Drug administration dosage, nephrotoxicity study, and pharmacokinetic analysis.

The clinically recommended doses of zidovudine and amikacin are 6 mg/kg/day and 15 mg/kg/day, respectively. The rationale for selecting zidovudine and amikacin doses was based on the recommended corresponding surface area dosage conversion factor (48). Therefore, the dosages of zidovudine and amikacin in the rats were 40 mg/kg and 100 mg/kg once daily, respectively.

Blood samples were taken from the tail of each rat at baseline and each day prior to dosing to determine serum creatinine levels. The samples were allowed to clot and centrifuged at 11,000 rpm for 15 min; then, the serum was drawn off and stored at −80°C until analysis. Serum creatinine level was analyzed with a clinical chemistry analyzer (Roche Pharmaceutical Ltd., Basel, Switzerland). Nephrotoxicity was defined as the doubling of the baseline creatinine level (49).

To determine the concentrations of zidovudine and amikacin in TCF, samples were taken from each rat at several time points (0, 0.5, 1,2, 4, 8, 12, and 24 h) on the last day of therapy. The samples were immediately centrifuged at 10,000 rpm for 10 min and then stored at −80°C until analysis. The concentrations of zidovudine and amikacin were analyzed through high-performance liquid chromatography and liquid chromatography-tandem mass spectrometry as previously described (50, 51).

### Efficacy of the antibiotic therapies.

The efficacy of the various drug regimens was quantitatively assessed by the total bacterial population in the TCF. Bacterial populations were quantified by plating the 10-fold serially diluted samples (100 µL) on Mueller-Hinton agar (MHA) plates. The bacterial colonies of each plate were counted after 24-h incubation at 35°C in ambient air. The detection limit of bacterial population was 1 log_10_ CFU/mL of TCF.

### Statistical analysis.

Statistical analysis was performed using GraphPad Prism 8.0.2 software. The *in vitro* data were obtained from three independent experiments on separate days, and all data are presented as means and standard deviations (SD). In the Galleria mellonella infection model studies and nephrotoxicity study, the survival rates and onset of nephrotoxicity were analyzed using the log-rank test. In the rat tissue cage infection model studies, differences in bacterial counts between the groups were analyzed using the Mann-Whitney U test. For each test, a *P* value of <0.05 indicated a statistically significant difference.

### Data availability.

The data that support the findings of this study are available within the article and its supplemental material.

## References

[1] Gupta N, Limbago BM, Patel JB, Kallen AJ. 2011. Carbapenem-resistant Enterobacteriaceae: epidemiology and prevention. Clin Infect Dis 53:60–67. doi:10.1093/cid/cir202. 21653305

[2] Zhu J, Sun L, Ding B, Yang Y, Xu X, Liu W, Zhu D, Yang F, Zhang H, Hu F. 2016. Outbreak of sNDM-1-producing Klebsiella pneumoniae ST76 and ST37 isolates in neonates. Eur J Clin Microbiol Infect Dis 35:611–618. doi:10.1007/s10096-016-2578-z. 26803822

[3] Hsu LY, Apisarnthanarak A, Khan E, Suwantarat N, Ghafur A, Tambyah PA. 2017. Carbapenem-resistant Acinetobacter baumannii and Enterobacteriaceae in South and Southeast Asia. Clin Microbiol Rev 30:1–22. doi:10.1128/CMR.00042-16. 27795305 PMC5217790

[4] Li J, Nation RL, Turnidge JD, Milne RW, Coulthard K, Rayner CR, Paterson DL. 2006. Colistin: the re-emerging antibiotic for multidrug-resistant Gram-negative bacterial infections. Lancet Infect Dis 6:589–601. doi:10.1016/S1473-3099(06)70580-1. 16931410

[5] Liu Y-Y, Wang Y, Walsh TR, Yi L-X, Zhang R, Spencer J, Doi Y, Tian G, Dong B, Huang X, Yu L-F, Gu D, Ren H, Chen X, Lv L, He D, Zhou H, Liang Z, Liu J-H, Shen J. 2016. Emergence of plasmid-mediated colistin resistance mechanism MCR-1 in animals and human beings in China: a microbiological and molecular biological study. Lancet Infect Dis 16:161–168. doi:10.1016/S1473-3099(15)00424-7. 26603172

[6] Coates AR, Hu Y. 2007. Novel approaches to developing new antibiotics for bacterial infections. Br J Pharmacol 152:1147–1154. doi:10.1038/sj.bjp.0707432. 17704820 PMC2189988

[7] Peyclit L, Baron SA, Rolain JM. 2019. Drug repurposing to fight colistin and carbapenem-resistant bacteria. Front Cell Infect Microbiol 9:193. doi:10.3389/fcimb.2019.00193. 31245302 PMC6579884

[8] Rafailidis PI, Falagas ME. 2014. Options for treating carbapenem-resistant Enterobacteriaceae. Curr Opin Infect Dis 27:479–483. doi:10.1097/QCO.0000000000000109. 25259809

[9] Ezzell C. 1987. AZT given the green light for clinical treatment of AIDS. Nature 326:430. doi:10.1038/326430b0. 3470603

[10] Elwell LP, Ferone R, Freeman GA, Fyfe JA, Hill JA, Ray PH, Richards CA, Singer SC, Knick VB, Rideout JL. 1987. Antibacterial activity and mechanism of action of 3'-azido-3'-deoxythymidine (BW A509U). Antimicrob Agents Chemother 31:274–280. doi:10.1128/AAC.31.2.274. 3551832 PMC174705

[11] Peyclit L, Baron SA, Yousfi H, Rolain JM. 2018. Zidovudine: a salvage therapy for mcr-1 plasmid-mediated colistin-resistant bacterial infections? Int J Antimicrob Agents 52:11–13. doi:10.1016/j.ijantimicag.2018.03.012. 29580929

[12] Keith BR, White G, Wilson HR. 1989. In vivo efficacy of zidovudine (3'-azido-3'-deoxythymidine) in experimental gram-negative-bacterial infections. Antimicrob Agents Chemother 33:479–483. doi:10.1128/AAC.33.4.479. 2658792 PMC172464

[13] Hu Y, Liu Y, Coates A. 2019. Azidothymidine produces synergistic activity in combination with colistin against antibiotic-resistant Enterobacteriaceae. Antimicrob Agents Chemother 63:e01630-18. doi:10.1128/AAC.01630-18. 30373798 PMC6325217

[14] Chang YT, Yang TY, Lu PL, Lin SY, Wang LC, Wang SF, Hsieh YJ, Tseng SP. 2020. Combination of colistin and azidothymidine demonstrates synergistic activity against colistin-resistant, carbapenem-resistant Klebsiella pneumoniae. Microorganisms 8:1964. doi:10.3390/microorganisms8121964. 33322306 PMC7764370

[15] Ng SMS, Sioson JSP, Yap JM, Ng FM, Ching HSV, Teo JWP, Jureen R, Hill J, Chia CSB. 2018. Repurposing zidovudine in combination with tigecycline for treating carbapenem-resistant Enterobacteriaceae infections. Eur J Clin Microbiol Infect Dis 37:141–148. doi:10.1007/s10096-017-3114-5. 29019016

[16] Antonello RM, Di Bella S, Betts J, La Ragione R, Bressan R, Principe L, Morabito S, Gigliucci F, Tozzoli R, Busetti M, Knezevich A, Furlanis L, Fontana F, Luzzaro F, Luzzati R, Lagatolla C. 2021. Zidovudine in synergistic combination with fosfomycin: an in vitro and in vivo evaluation against multidrug-resistant Enterobacterales. Int J Antimicrob Agents 58:106362. doi:10.1016/j.ijantimicag.2021.106362. 34010710

[17] Hu Y, Coates A. 2021. Zidovudine enhances activity of carbapenems against NDM-1-producing Enterobacteriaceae. J Antimicrob Chemother 76:2302–2305. doi:10.1093/jac/dkab184. 34120178 PMC8654596

[18] Wambaugh MA, Shakya VPS, Lewis AJ, Mulvey MA, Brown JCS. 2017. High-throughput identification and rational design of synergistic small-molecule pairs for combating and bypassing antibiotic resistance. PLoS Biol 15:e2001644. doi:10.1371/journal.pbio.2001644. 28632788 PMC5478098

[19] Doleans-Jordheim A, Bergeron E, Bereyziat F, Ben-Larbi S, Dumitrescu O, Mazoyer MA, Morfin F, Dumontet C, Freney J, Jordheim LP. 2011. Zidovudine (AZT) has a bactericidal effect on enterobacteria and induces genetic modifications in resistant strains. Eur J Clin Microbiol Infect Dis 30:1249–1256. doi:10.1007/s10096-011-1220-3. 21494911

[20] Diriba K, Awulachew E, Gemede A, Anja A. 2021. The magnitude of extended-spectrum beta-lactamase-producing Enterobacteriaceae from clinical samples in Ethiopia: a systematic review and meta-analysis. Access Microbiol 3:000195. doi:10.1099/acmi.0.000195. 34151151 PMC8209701

[21] Ramirez MS, Tolmasky ME. 2017. Amikacin: uses, resistance, and prospects for inhibition. Molecules 22:2267. doi:10.3390/molecules22122267. 29257114 PMC5889950

[22] Blum MR, Liao SH, Good SS, de Miranda P. 1988. Pharmacokinetics and bioavailability of zidovudine in humans. Am J Med 85:189–194. 3165603

[23] Klecker RW, Jr, Collins JM, Yarchoan R, Thomas R, Jenkins JF, Broder S, Myers CE. 1987. Plasma and cerebrospinal fluid pharmacokinetics of 3'-azido-3'-deoxythymidine: a novel pyrimidine analog with potential application for the treatment of patients with AIDS and related diseases. Clin Pharmacol Ther 41:407–412. doi:10.1038/clpt.1987.49. 3549120

[24] Beach JW. 1998. Chemotherapeutic agents for human immunodeficiency virus infection: mechanism of action, pharmacokinetics, metabolism, and adverse reactions. Clin Ther 20:2–25. doi:10.1016/S0149-2918(98)80031-3. 9522101

[25] Pickus OB. 1988. Overdose of zidovudine. N Engl J Med 318:1206. doi:10.1056/NEJM198805053181817. 3163102

[26] Ayers KM. 1988. Preclinical toxicology of zidovudine. Am J Med 85:186–188. 3044084

[27] Yarchoan R, Klecker RW, Weinhold KJ, Markham PD, Lyerly HK, Durack DT, Gelmann E, Lehrman SN, Blum RM, Barry DW. 1986. Administration of 3'-azido-3'-deoxythymidine, an inhibitor of HTLV-III/LAV replication, to patients with AIDS or AIDS-related complex. Lancet i:575–580. doi:10.1016/s0140-6736(86)92808-4. 2869302

[28] Wattanagoon Y, Na Bangchang K, Hoggard PG, Khoo SH, Gibbons SE, Phiboonbhanakit D, Karbwang J, Back DJ. 2000. Pharmacokinetics of zidovudine phosphorylation in human immunodeficiency virus-positive thai patients and healthy volunteers. Antimicrob Agents Chemother 44:1986–1989. doi:10.1128/AAC.44.7.1986-1989.2000. 10858368 PMC89999

[29] Burger DM, Meenhorst PL, ten Napel CH, Mulder JW, Neef C, Koks CH, Bult A, Beijnen JH. 1994. Pharmacokinetic variability of zidovudine in HIV-infected individuals: subgroup analysis and drug interactions. AIDS 8:1683–1689. doi:10.1097/00002030-199412000-00007. 7888117

[30] Hoetelmans RM, Burger DM, Meenhorst PL, Beijnen JH. 1996. Pharmacokinetic individualisation of zidovudine therapy. Current state of pharmacokinetic-pharmacodynamic relationships. Clin Pharmacokinet 30:314–327. doi:10.2165/00003088-199630040-00004. 8983861

[31] Wilde MI, Langtry HD. 1993. Zidovudine. An update of its pharmacodynamic and pharmacokinetic properties, and therapeutic efficacy. Drugs 46:515–578. doi:10.2165/00003495-199346030-00010. 7693435

[32] Magnet S, Blanchard JS. 2005. Molecular insights into aminoglycoside action and resistance. Chem Rev 105:477–498. doi:10.1021/cr0301088. 15700953

[33] Wachino J, Arakawa Y. 2012. Exogenously acquired 16S rRNA methyltransferases found in aminoglycoside-resistant pathogenic Gram-negative bacteria: an update. Drug Resist Updat 15:133–148. doi:10.1016/j.drup.2012.05.001. 22673098

[34] Ramirez MS, Tolmasky ME. 2010. Aminoglycoside modifying enzymes. Drug Resist Updat 13:151–171. doi:10.1016/j.drup.2010.08.003. 20833577 PMC2992599

[35] Hu X, Xu B, Yang Y, Liu D, Yang M, Wang J, Shen H, Zhou X, Ma X. 2013. A high throughput multiplex PCR assay for simultaneous detection of seven aminoglycoside-resistance genes in Enterobacteriaceae. BMC Microbiol 13:58. doi:10.1186/1471-2180-13-58. 23497180 PMC3637108

[36] Cihlar T, Ray AS. 2010. Nucleoside and nucleotide HIV reverse transcriptase inhibitors: 25 years after zidovudine. Antiviral Res 85:39–58. doi:10.1016/j.antiviral.2009.09.014. 19887088

[37] Liu Y, Jia Y, Yang K, Li R, Xiao X, Wang Z. 2020. Anti-HIV agent azidothymidine decreases Tet(X)-mediated bacterial resistance to tigecycline in Escherichia coli. Commun Biol 3:162. doi:10.1038/s42003-020-0877-5. 32246108 PMC7125129

[38] CLSI. 2018. Performance standards for antimicrobial susceptibility testing, document M100, 28th ed. CLSI, Wayne, PA.

[39] Yeganeh Sefidan F, Mohammadzadeh-Asl Y, Ghotaslou R. 2019. High-level resistance to aminoglycosides due to 16S rRNA methylation in Enterobacteriaceae isolates. Microb Drug Resist 25:1261–1265. doi:10.1089/mdr.2018.0171. 31211656

[40] Bercot B, Poirel L, Nordmann P. 2011. Updated multiplex polymerase chain reaction for detection of 16S rRNA methylases: high prevalence among NDM-1 producers. Diagn Microbiol Infect Dis 71:442–445. doi:10.1016/j.diagmicrobio.2011.08.016. 22000158

[41] Park CH, Robicsek A, Jacoby GA, Sahm D, Hooper DC. 2006. Prevalence in the United States of aac(6')-Ib-cr encoding a ciprofloxacin-modifying enzyme. Antimicrob Agents Chemother 50:3953–3955. doi:10.1128/AAC.00915-06. 16954321 PMC1635235

[42] Odds FC. 2003. Synergy, antagonism, and what the chequerboard puts between them. J Antimicrob Chemother 52:1. doi:10.1093/jac/dkg301. 12805255

[43] Doern CD. 2014. When does 2 plus 2 equal 5? A review of antimicrobial synergy testing. J Clin Microbiol 52:4124–4128. doi:10.1128/JCM.01121-14. 24920779 PMC4313275

[44] White RL, Burgess DS, Manduru M, Bosso JA. 1996. Comparison of three different in vitro methods of detecting synergy: time-kill, checkerboard, and E test. Antimicrob Agents Chemother 40:1914–1918. doi:10.1128/AAC.40.8.1914. 8843303 PMC163439

[45] Betts J, Nagel C, Schatzschneider U, Poole R, La Ragione RM. 2017. Antimicrobial activity of carbon monoxide-releasing molecule [Mn(CO)_3_(tpa-κ^3^N)]Br versus multidrug-resistant isolates of avian pathogenic Escherichia coli and its synergy with colistin. PLoS One 12:e0186359. doi:10.1371/journal.pone.0186359. 29040287 PMC5645124

[46] Ni W, Yang D, Guan J, Xi W, Zhou D, Zhao L, Cui J, Xu Y, Gao Z, Liu Y. 2021. In vitro and in vivo synergistic effects of tigecycline combined with aminoglycosides on carbapenem-resistant Klebsiella pneumoniae. J Antimicrob Chemother 76:2097–2105. doi:10.1093/jac/dkab122. 33860309

[47] Ruppen C, Mercier T, Grandgirard D, Leib SL, El Haj C, Murillo O, Decosterd L, Sendi P. 2018. Is penicillin plus gentamicin synergistic against sessile group B streptococcal isolates? An in vivo study with an experimental model of foreign-body infection. Front Microbiol 9:919. doi:10.3389/fmicb.2018.00919. 29867830 PMC5962661

[48] Nair AB, Jacob S. 2016. A simple practice guide for dose conversion between animals and human. J Basic Clin Pharm 7:27–31. doi:10.4103/0976-0105.177703. 27057123 PMC4804402

[49] Chan K, Ledesma KR, Wang W, Tam VH. 2020. Characterization of amikacin drug exposure and nephrotoxicity in an animal model. Antimicrob Agents Chemother 64:e00859-20. doi:10.1128/AAC.00859-20. 32571819 PMC7449196

[50] Ni W, Yang D, Mei H, Zhao J, Liang B, Bai N, Chai D, Cui J, Wang R, Liu Y. 2017. Penetration of ciprofloxacin and amikacin into the alveolar epithelial lining fluid of rats with pulmonary fibrosis. Antimicrob Agents Chemother 61:e01936-16. doi:10.1128/AAC.01936-16. 28115351 PMC5365682

[51] Alnouti Y, White CA, Bartlett MG. 2004. Simultaneous determination of zidovudine and lamivudine from rat plasma, amniotic fluid and tissues by HPLC. Biomed Chromatogr 18:641–647. doi:10.1002/bmc.367. 15386504

