# Supplementary material for: Efficacy of Zidovudine-Amikacin Combination Therapy In Vitro and in a Rat Tissue Cage Infection Model against Amikacin-Resistant, Multidrug-Resistant Enterobacteriales
Source: Microbiol Spectr. 2023 Mar 22;11(2):e04843-22. doi: 10.1128/spectrum.04843-22 (PMC10101109; doi:10.1128/spectrum.04843-22)
Supplement: Supplemental file 1 — Supplemental material. Download spectrum.04843-22-s0001.pdf, PDF file, 0.3 MB [file spectrum.04843-22-s0001.pdf]

Table S1. Resistance profiles and genetic determinants of tested strains.

STRAINS	NAME [a]	RESISTANCE GENES	ZDV+AMK FICI	NON SUSCEPTIBLE TO [b] CST	TIG	AMK	TOB	IPM	MEM	ATM	CTZ	CPM	AMC	PIP	CIP	SXT
K. pneumoniae	CN-1	rmtB	0.156	■	■	■	■	■	■	■	■	■	■	■	■	■
	CN-2	-	0.252			■	■	■	■	■	■	■	■	■	■	■
	CN-3	-	0.066	■	■	■	■	■	■	■	■	■	■	■	■	■
	CN-4	aac(6')-Ib	0.281		■	■	■	■	■	■	■	■	■	■	■	■
	CN-5	aac(6')-Ib	0.252		■	■	■	■	■	■	■	■	■	■	■	■
	CN-6	aac(6')-Ib	0.313			■	■	■	■	■	■	■	■	■	■	■
	CN-7	aac(6')-Ib, armA	1	■	■	■	■	■	■	■	■	■	■	■	■	■
	CN-8	aac(6')-Ib	0.266			■	■	■	■	■	■	■	■	■	■	■
	CN-9	-	0.125			■	■	■	■	■	■	■	■	■	■	■
	CN-10	aac(6')-Ib, rmtB, rmtC	0.07		■	■	■	■	■	■	■	■	■	■	■	■
	AF-1	aac(6')-Ib, rmtB	0.625	■	■	■	■	■	■	■	■	■	■	■	■	■
	AF-2	aac(6')-Ib	0.5	■	■	■	■	■	■	■	■	■	■	■	■	■
	AF-3	aac(6')-Ib, rmtC	0.033			■	■	■	■	■	■	■	■	■	■	■
	AF-4	aac(6')-Ib, rmtC	0.375			■	■	■	■	■	■	■	■	■	■	■
	AF-5	rmtB	0.625		■	■	■	■	■	■	■	■	■	■	■	■
	AF-6	aac(6')-Ib	0.035		■	■	■	■	■	■	■	■	■	■	■	■
	AF-7	-	0.127	■	■	■	■	■	■	■	■	■	■	■	■	■
	AF-8	rmtB	0.266			■	■	■	■	■	■	■	■	■	■	■
	AF-9	rmtB	0.252		■	■	■	■	■	■	■	■	■	■	■	■
	AF-10	aac(6')-Ib	0.516	■	■	■	■	■	■	■	■	■	■	■	■	■
	AF-11	aac(6')-Ib	0.065		■	■	■	■	■	■	■	■	■	■	■	■
	AF-12	rmtB	0.516	■	■	■	■	■	■	■	■	■	■	■	■	■
E. coli	CN-11	aac(6')-Ib, rmtB	0.5			■	■	■	■	■	■	■	■	■	■	■
	CN-12	aac(6')-Ib, rmtB	0.141			■	■	■	■	■	■	■	■	■	■	■
	CN-13	rmtB	1		■	■	■	■	■	■	■	■	■	■	■	■
	CN-14	rmtB	1			■	■	■	■	■	■	■	■	■	■	■
	CN-15	rmtB	0.531			■	■	■	■	■	■	■	■	■	■	■
	CN-16	aac(6')-Ib, rmtB	0.375		■	■	■	■	■	■	■	■	■	■	■	■
	CN-17	aac(6')-Ib	0.625		■	■	■	■	■	■	■	■	■	■	■	■
	CN-18	rmtB	1			■	■	■	■	■	■	■	■	■	■	■
	CN-19	aac(6')-Ib,armA, rmtB	0.5		■	■	■	■	■	■	■	■	■	■	■	■
	CN-20	rmtB	0.625			■	■	■	■	■	■	■	■	■	■	■
	CN-21	aac(6')-Ib	0.141			■	■	■	■	■	■	■	■	■	■	■
	AF-13	aac(6')-Ib, armA	0.281		■	■	■	■	■	■	■	■	■	■	■	■
	AF-14	rmtB	0.252			■	■	■	■	■	■	■	■	■	■	■
	AF-15	aac(6')-Ib,armA, rmtB	0.188			■	■	■	■	■	■	■	■	■	■	■
	AF-16	-	0.188		■	■	■	■	■	■	■	■	■	■	■	■
	AF-17	-	0.156		■	■	■	■	■	■	■	■	■	■	■	■
	AF-18	rmtB	0.258			■	■	■	■	■	■	■	■	■	■	■
	AF-19	aac(6')-Ib, rmtB	0.281			■	■	■	■	■	■	■	■	■	■	■
	AF-20	rmtB	0.141		■	■	■	■	■	■	■	■	■	■	■	■
	AF-21	-	0.188			■	■	■	■	■	■	■	■	■	■	■
	AF-22	aac(6')-Ib, armA	0.188			■	■	■	■	■	■	■	■	■	■	■
	AF-23	rmtB	0.5			■	■	■	■	■	■	■	■	■	■	■
	AF-24	rmtB	1		■	■	■	■	■	■	■	■	■	■	■	■

	AF-25	aac(6’)-Ib	0.156													
	AF-26	rmtB	0.5													
	AF-27	rmtB	1													
	AF-28	rmtB	0.25													
	AF-29	rmtB	0.516													
	AF-30	rmtB	0.188													
	AF-31	aac(6’)-Ib	0.502													
	AF-32	rmtB	0.375													

[a]CN: XuZhou Central Hospital origin; AF: the Affiliated Hospital of Xuzhou Medical University origin

[b] CST: colistin; TIG: tigecycline; AMK: amikacin; TOB: tobramycin; IPM: imipenem; MEM: meropenem; ATM: aztreonam; CTZ: ceftazidime; CPM: cefepime; AMC: amoxicillin-clavulanate; PIP: piperacillin-tazobactam; CIP: ciprofloxacin; SXT: trimethoprim/sulfamethoxazole.

The white and gray squares represent susceptibility and non-susceptibility to above drugs respectively.

Table S2. Oligonucleotides used in this study.

GENES	PRIMER	SEQUENCE 5’-3’	PRODUCT SIZE (BP)	REFERENCE
16S rRNA methylase genes	armA	F: ATTTTAGATTTTGGTTGTGGC	101	1
		R: ATCTCAGCTCTATCAATATCG		
	rmtA	F: AAACTATTCCGCATGGTTC	88	1
		R: TCATGTACACAAGCTCTTTCC		
	rmtB	F: ACTTTTACAATCCCTCAATAC	171	1
		R: AAGTATATAAGTTCTGTTCCG		
	rmtC	F: CAGGGGTTCCAACAAGT	246	1
		R:AGAGTATATAGCTTGAACATAAGTAGA		
	rmtD	F: GGAAAAGGACGTGGACA	171	1
		R: TCCATCGATTCCACAGG		
	npmA	F: GGGCTATCTAATGTGGTG	229	1
		R: TTTTTATTTCCGCTTCTTCGT		
aminoglycoside modifying enzymes genes	aac(6')-Ib	F: TTGCGATGCTCTATGAGTGGCTA	482	2
		R: CTCGAATGCCTGGCGTGTTT		
	aac(6')-II	F: CGACCATTTCATGTCC	542	3
		R: GAAGGCTTGTCGTGTTT		

1. Bercot B, Poirel L, Nordmann P. Updated multiplex polymerase chain reaction for detection of 16S rRNA methylases: high prevalence among NDM-1 producers. Diagn Microbiol Infect Dis. Dec 2011;71(4):442-445.

2.Park CH, Robicsek A, Jacoby GA, Sahm D, Hooper DC. Prevalence in the United States of aac(6')-Ib-cr encoding a ciprofloxacin-modifying enzyme. Antimicrob Agents Chemother. Nov 2006;50(11):3953-3955.

3.Hu X, Xu B, Yang Y, et al. A high throughput multiplex PCR assay for simultaneous detection of seven aminoglycoside-resistance genes in Enterobacteriaceae. BMC Microbiol. Mar 14 2013;13:58.

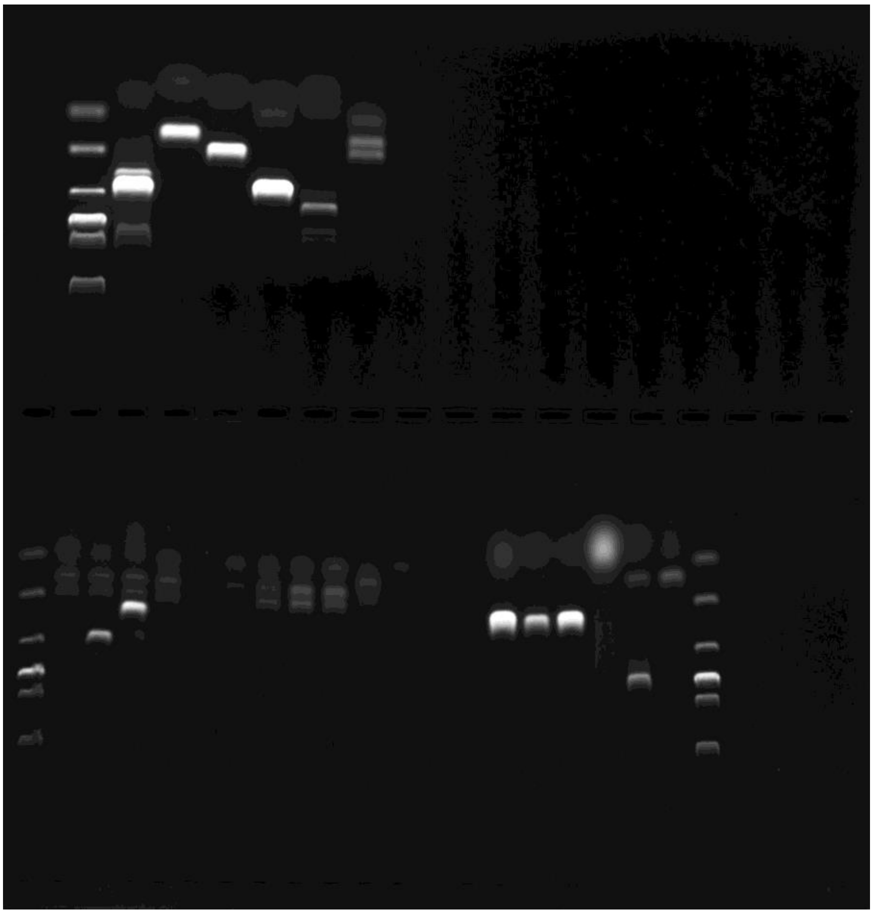

Figure S1: Representative DNA fingerprint pattern of Enterobacteriaceae isolates genotyped by PCR

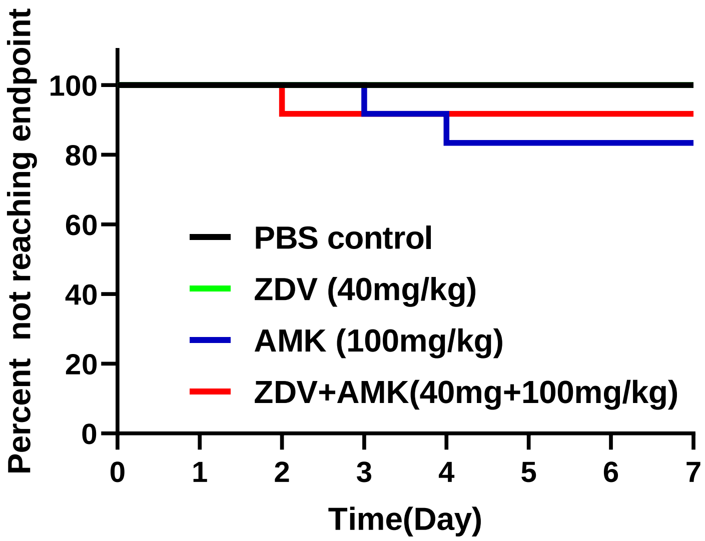

Figure S2: Time to onset of nephrotoxicity. n = 12 per group, P = 0.289.
